# Improving Healthy Aging by Monitoring Patients’ Lifestyle through a Wearable Device: Results of a Feasibility Study

**DOI:** 10.3390/ijerph18189806

**Published:** 2021-09-17

**Authors:** Maria Fioranzato, Rosanna Irene Comoretto, Corrado Lanera, Lamberto Pressato, Giuseppe Palmisano, Luca Barbacane, Dario Gregori

**Affiliations:** 1 Unit of Biostatistics, Epidemiology and Public Health, Department of Cardiac, Thoracic, Vascular Sciences and Public Health, University of Padova, 35131 Padova, Italy; maria.fioranzato@gmail.com (M.F.); rosannairene.comoretto@unito.it (R.I.C.); corrado.lanera@unipd.it (C.L.); 2 Department of Public Health and Pediatrics, University of Turin, 10126 Turin, Italy; 3 Group of Primary Care of Vigonovo, Unità Locale Socio-Sanitaria ULSS 3 Serenissima, 30030 Vigonovo, Italy; lamberto.pressato@gmail.com; 4 Group of Primary Care of Quarto D’Altino, Unità Locale Socio-Sanitaria ULSS 3 Serenissima, 30020 Quarto d’Altino, Italy; dott.palmisano@libero.it; 5 Group of Primary Care of Martellago, Unità Locale Socio-Sanitaria ULSS 3 Serenissima, 30030 Martellago, Italy; luca.barbacane@aulss3.veneto.it

**Keywords:** wearable device, feasibility study, lifestyle monitoring

## Abstract

Population aging is related to a huge growth in healthcare and welfare costs. Therefore, wearable devices could be strategic for minimizing years of disability in old age and monitoring patients’ lifestyles and health. The purpose of this study was to assess the feasibility of using smart devices to monitor patients’ physical activity in a primary care setting. To assess the acceptance of this novel technology from the point of view of both patients and healthcare professionals, two questionnaires (one paper-based and one ex-novo developed) were administered to 11 patients with type 2 diabetes mellitus and a non-compliant behavior towards the therapeutic indications of their general practitioner (GP). Seven participants would continue to use a wearable activity tracker to monitor their health. We observed that 75% of patients reported a device’s characteristics satisfaction level of over 80% of the total score assigned to this dimension. No differences were observed in the questionnaire’s scores between the two professionals categories (GPs and nurses). Three dimensions (equipment characteristics, subjective norm, perceived risks, perceived ease-of-use and facilitating conditions) correlated > 0.5 with the device’s acceptability level. Some weak correlations were observed between healthcare professionals’ perception and patients’ parameters, particularly between the dimensions of collaboration and web interface ease-of-use and patients’ median number of steps and hours of sleep. In conclusion, despite the limited number of subjects involved, a good acceptance level towards these non-medical devices was observed, according to both patients’ and healthcare professionals’ impressions.

## 1. Introduction

It is well known that the population is aging. Life expectancy is constantly increasing, not only in developed countries but also in developing ones. Therefore, aging is a phenomenon that involves the entire world population.

In the Italian context, Istat [1] data show that the men’s average lifespan will shift from 77.4 years in 2005 to 83.6 years in 2050, and for women, it will rise from 83.3 to 88.8 years. The amount of over 65 individuals, which today is the 20.3% of the population, will rise to over 32% in 2043 [2,3].

Nevertheless, if this new life expectancy is an achievement on one side, it has required, and it still does, significant structural changes in society, both on the economic level and social security, welfare, and healthcare.

Therefore, successful aging is of fundamental importance to preserve subjects’ good quality of life and limit healthcare costs. In this framework, a key concept is aging-in-place, representing the ability to continue living at home while maintaining personal independence, dignity, and social interaction [4,5,6]. In older people, a healthy lifestyle is particularly challenged due to the presence of concomitant chronic diseases. In this population, monitoring physical activity is a valuable parameter to define if people lead a healthy lifestyle to prevent chronic diseases or complications, or show early symptoms of these events [4].

Self-reporting is a straightforward tool for lifestyle and physical activity assessments through the completion of questionnaires, interviews, and surveys [5]. However, such an approach presents several limitations that make monitoring lifestyle difficult (e.g., recall and desirability biases). Recently, the increased availability of novel technologies such as smart devices and the rapidly growing interest in wearable devices have changed the approach to lifestyle monitoring [6]. They have been employed to monitor both lifestyle habits (physical activity, dietary habits, sleep) and clinical parameters (e.g., heart rate) in both healthy subjects and those affected by acute or chronic diseases [7,8]. It has also been reported that wearable motion detectors are the most promising technology enabling an automatic, continuous, and long-term assessment of subjects in their daily life environments [9,10]. In addition, lifestyle parameters can be shared with healthcare providers and insurance platforms to describe better the behavioral pattern and functional ability in high-risk subjects, thus providing important feedback regarding the overall health status of an individual and even prediction of potential adverse health events.

In 2012, the Veneto Region boosted the function of General Practitioners (GPs) [11], developing the Integrated Care Groups (ICG) [12]. In such structures, the coexistence of GPs, nurses, and assistants ensures that the patient is entirely handled, especially for promoting a healthy lifestyle and performing more straightforward tests, for overall monitoring of the health status, health education, and education for elders and their caregivers.

As the role of GPs in monitoring lifestyle is recognized to be of primary importance in assessing patient health needs, knowing clinical history, living habits, and risk factors, in this setting, wearable devices could be used as allies in patient assessment, allowing constant and complete remote monitoring of subjects’ lifestyle by health professionals.

However, only a few studies have explored the acceptance of the different types of smart devices in the older population [13,14,15,16], but the short duration of such studies (from one to a few weeks) has left the investigation of this novel technology limited to the first impression [17]. Short-term technology acceptance may not be correlated to long-term acceptance, because it seems that the use of wearables devices tends to drop after the first few weeks of ownership [18]. Furthermore, most existing studies [14,15,16] examined the acceptance level based on the technology acceptance model (TAM) [17].

The purpose of this study was to assess the feasibility of using smart wearable devices (such as smartwatches) in order to monitor patients’ physical activity in a primary care setting.

Therefore, the main aim was to evaluate the acceptance of wearable activity trackers in a population of active older adults with diabetes in their daily life setting and assess patients’ compliance in wearing the device in a medium-term period. Secondarily, the study would evaluate GPs’ and nurses’ ability to interact with monitoring systems and their technology acceptance level.

## 2. Materials and Methods

### 2.1. Sample and Setting

The study involved three centers in the territory of Local Health Unit 3 “Serenissima”, in the Veneto Region: the integrated primary care groups of Martellago and Vigonovo (Local Health District of Mirano-Dolo) and the integrated primary care group of Quarto ‘d’Altino (Local Health District of Venezia Terraferma, Marcon e Quarto d’Altino).

A convenience sample of subjects living in this territory was recruited among those referred to each GP participating in the study. The inclusion criteria were age ≥60 years and <75 years, the presence of type 2 diabetes mellitus, and a non-compliant behavior towards the therapeutic indications of their GP (behavioral intervention-resistant subjects). Moreover, patients (or a caregiver living with them) had to own a smartphone and be reasonably independent in using it.

### 2.2. Study Design

In this multicenter prospective observational feasibility study, a convenience sampling technique was used to recruit 11 older adults with diabetes, based on a strategy adopted by Puri et al. [13].

A baseline data collection about socio-demographic characteristics (gender, age, marital status, educational level, working status, living conditions), anthropometric characteristics (height and weight to calculate body mass index (BMI)), the trend of the glycated hemoglobin in the last two years (with half-yearly intervals) and the on-going diabetic treatment (oral hypoglycemic agent or insulin) was performed. Every patient was given a smartwatch activity tracker vívoactive^®^ 3 device by Garmin© with the information concerning its use. At the same time, the Garmin Connect© app (connected to the device via Bluetooth^®^) was installed on their smartphone. Hence, a personal account was set up on the Garmin Connect platform, where data collected by the device (numbers of steps, sleep duration, heart rate, etc.) were available to patients and GPs/nurses. Patients were asked to wear the device 24 h a day, seven days a week, until the end of the study (set up at seven weeks) except for the moments when it would be necessary to recharge the device.

At the end of the follow-up (after seven weeks), patients and the medical staff involved in the study completed two different questionnaires, one specifically for the patients, the other for the medical staff. At the same time, data were collected from the Garmin Connect platform to assess compliance with this new technology.

All patients involved gave written informed consent to participate in the study and share their personal Garmin account with GPs and nurses.

### 2.3. Technology Acceptance and Interaction Assessment

For patients’ technology acceptance, a 31-item, 5-point Likert scale, and an additional six multiple-choice items, self-reported, paper-based survey for elders was adapted from Puri et al. [13], based on the fundamental dimensions that influence the user acceptance of technology from TAM [17] and the sensor acceptance model [19]. The investigated key dimensions for wearable activity tracker acceptance were perceived usefulness, perceived ease-of-use, privacy concerns, perceived risks, facilitating conditions, and equipment characteristics. The description of the technology acceptance survey, including each dimension and related questions, is reported in Table S1.

GPs and nurses were also given an ad hoc survey that evaluated (i) their ability to interact with the technology adopted and (ii) the actual possibility of intervention on patients in changing lifestyle strategies. Additionally, in this case, questionnaire’s items were grouped into four different dimensions: clinical usefulness, management usefulness, web interface ease-of-use, and collaboration (between professionals and patients). Description of the technology interaction survey, including each dimension and corresponding items, is reported in Table S2. Both questionnaires are fully reported in Tables S3 and S4.

### 2.4. Statistical Analysis

The study was designed to assess the feasibility of wearable device usage in monitoring patients’ health status in their daily living setting.

Baseline data are reported as median (I and III quartiles) for continuous variables, and percentages (absolute numbers), for qualitative variables. Wilcoxon and Kruskal–Wallis tests were performed for continuous variables and Pearson’s chi-square test for categorical ones.

The acceptance of the wearable activity tracker was measured by question number 33: “Would you use the device you used to continue to monitor or track your physical activity or health?” The Kruskal–Wallis test was used to test for the differences in the technology acceptance/interaction questionnaires between two groups of patients (based on the educational level) and professionals (nurses vs. physicians). Similarly, the same test was used to test for the differences in data collected by the device between the two levels of acceptability. Third, Spearman’s rho (with relative 95% confidence intervals) was calculated to test whether a correlation between participants’ responses to the Likert scale questions and user acceptance exists. Finally, Spearman’s rho was also used to assess preliminarily the existence of a relationship between the professionals’ impressions and patients’ data collected by the device; health professionals and patients were linked based on center, and patients’ median values of data collected by the device (wearing time, sleep hours, steps and heart rate) were correlated to professionals’ scores.

The analyses were performed using the R System [20] with the rms package [21].

## 3. Results

Eleven participants were enrolled in the study. The number of participants actively wearing the smart wristbands during the follow-up was ten because one subject left the study in the first week. However, he filled in the questionnaire at the end of the follow-up.

Socio-demographic data and baseline characteristics of the whole study sample are described in Table 1. Most subjects were men (10/11, 91%), and 64% (7/11) have a secondary school education. During the study, only one participant used his caregiver’s smartphone while the other ten (91%) personally owned a smartphone and could use it independently. The participants used the devices for 49 days (seven weeks), except those who dropped out of the study early. Device’s data collections show the smartwatches were used more when the participants were awake because most of them removed them before sleeping. Only two patients declared to use a wearable activity tracker to monitor their health (on their watch or the website). Overall, the wearable activity trackers had a moderate level of acceptance: 7/11 participants would continue to use the wearable activity tracker to monitor their health (64%). No difference in age was detected between patients more prone to accept the device and those who were not (*p* = 0.534).

### 3.1. Technology Acceptance Questionnaire (Patients)

We observed that 75% of patients reported a device’s characteristics satisfaction level of over 80% of the total score assigned to this dimension. According to subjects’ educational level (primary or secondary school education), there were no differences in questionnaire’s scores in almost all dimensions (Table 2, Panel A), except for the perceived risk dimension (*p* = 0.033). The score in this dimension was higher for subjects with a secondary school education than those with primary school one.

**Figure 1:**
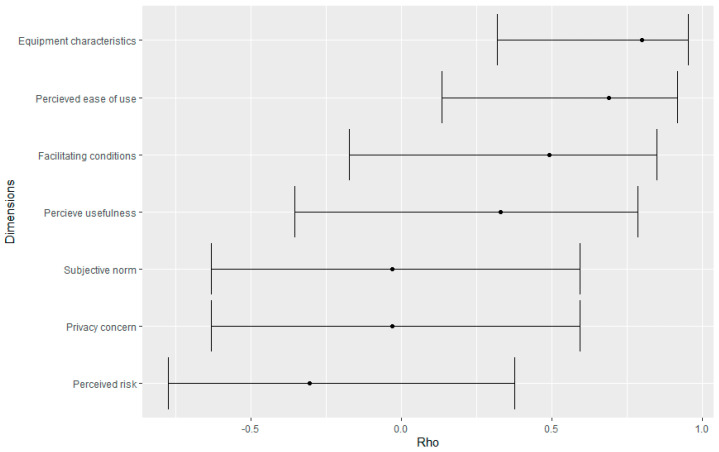
Correlation between the questionnaire’s dimensions and device’s acceptability level (represented by the response to question #33).

Figure 1 shows the correlation between the questionnaire’s dimensions and the acceptability of the device. Three dimensions (equipment characteristics, perceived ease-of-use and facilitating conditions) seem to be more correlated than the remaining four (perceived usefulness, subjective norm, privacy concerns, and perceived risks) with the acceptability level.

Table 3 shows the distribution of patients’ data collected by the device and their acceptance level (for the 10 patients that ended the study). No differences were observed between the two groups, but subjects more prone to accept the device had an overall wearing time higher than subjects that did not accept the device.

### 3.2. Technology Interaction Questionnaire (Professionals)

Six professionals were involved in the study (three GPs and three nurses). No significant differences were observed between the two groups in dimensions’ scores (Table 2, Panel B).

**Figure 2:**
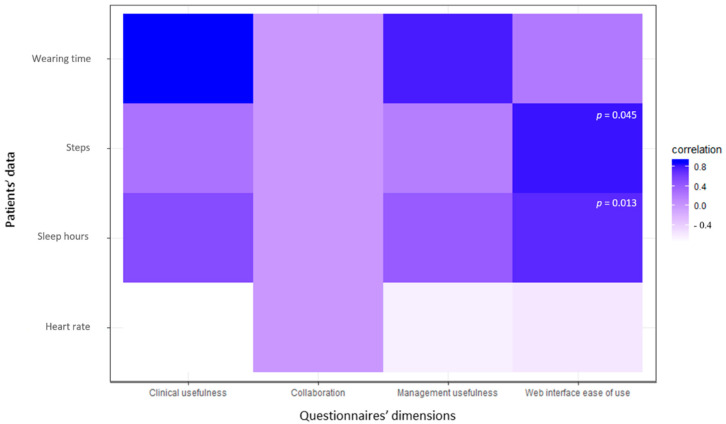
Correlation between professionals’ impressions and patients’ data.

Observing the correlation between professionals’ impressions (i.e., professionals answers to questionnaire items grouped in the four dimensions) and patients’ data collected by the devices, all dimensions seem to be inversely correlated with the median heart rate and, to a lesser extent, with the wearing time (Figure 2). Conversely, all dimensions are more correlated with the other patients’ data. In particular, we can also observe a good correlation (>0.5) between the dimensions of collaboration and web interface ease-of-use and patients’ median hours of sleep. Furthermore, there is a weak positive correlation between professionals’ questionnaire dimensions and patients’ median number of steps. The only two correlation coefficients which are statistically significant are those between web interface ease-of-use and sleep hours (*p* = 0.013) and number of steps (*p* = 0.045).

## 4. Discussion

This study aimed to evaluate the feasibility of using a non-medicalized device to monitor elderly diabetic patients in a primary care setting. Unlike previous studies, in this analysis, the point of view of both patients and healthcare professionals were considered: the device acceptance by the former and the perception of the usefulness by the latter.

The ten patients that completed the study wore the device for seven weeks. To our best knowledge, there are no literature studies explicitly evaluating the device’s acceptance for such a long time (more than 2 to 3 weeks). Additionally, it has been shown that in most cases, the use of the device falls drastically after 15 days of use [22]. In our case, the patients maintained constant use during all the weeks. Most of the participants were frequent computer and smartphone users and had high awareness of wearable activity trackers. However, the only patient who left the study in the first week filled in the questionnaire as his/her opinion was also considered essential to assess the device’s acceptability.

Overall, participants showed a good level of acceptance of the device (a median score of 27.2 (IQR 25.5–29.5) for the perception of device characteristics). Seven of them declared that they would keep using it to monitor their health status. Although they were less exposed to such wearable technology, we could argue that they felt this tracking was valuable and safe.

From the statistical analysis of the given answers, it emerges that the device’s features seemed to have fulfilled the participants’ expectations. The battery life encountered a good level of satisfaction for eight of the people involved in the study, and also for the other features, there was a good response. Nevertheless, it is not possible to notice a correlation between the satisfaction towards the device features and its acceptance by patients. This aspect remains an open field to be explored with future studies.

The analysis of this study showed that five of the subjects involved were highly satisfied with the level of privacy offered by this kind of monitoring. Moreover, no one indicated a disagreement with the following statement: “I feel safe about the fact that data about my health status are shared with the devices’ producers, as long as they are shared anonymously.” This result can be partially justified by the excellent information given to participants at the beginning of the study. In addition, the fact that data collected were stored in an anonymous database of a company outside the healthcare system seemed to play a crucial role. From the literature analysis, privacy appears to be a fundamental element in accepting wearable devices [13].

Conversely, some studies highlighted that, despite uncertainties over privacy implications, when the technology is perceived as a useful tool [23] or as a tool that allows maintaining physical and social autonomy [24], elderly subjects are more willing to share information and compromise their privacy. Even though a direct positive correlation between the acceptance of the device and the level of satisfaction with privacy issues has not been found (Figure 1, rho < 0), this remains an important topic to be further examined. It could also be investigated if several factors such as excellent initial information, the data collection organization, or other factors related to behaviors/cooperation with the healthcare professionals can influence the acceptance of devices from the point of view of privacy guarantee.

The second aim of this study was to explore the feasibility of using this type of monitoring by doctors and nurses. To the best of our knowledge, there are no studies on the perception of healthcare professionals about the use of non-medical devices to implement patient’s health.

Healthcare professionals have demonstrated a good acceptance of this type of monitoring, overall, options in disagreement or strong disagreement were not present in most answers. It has been found that monitoring physical activity and chronic patients’ health is helpful in clinical practice.

The use of a non-medical device seems to be a positive aspect of this study. All six professionals thought that patients feel more comfortable using this type of technology. Therefore, this technology could overcome stigmatization that has emerged using medical devices [25]. Additionally, it can also overcome the limits of self-reported data to assess subjects’ lifestyles [8,9,10].

In the statement “I think that the data collected from this type of monitoring are sufficient for the assistance given to this type of patient”, four professionals reported their disagreement. This is consistent with the clinical complexity of diabetic patients. However, professionals thought that data collected by the device could help in care programming, but they must be integrated with other purely clinical aspects that these devices cannot detect. In fact, through constant (also remotely) monitoring, the effects of interventions carried out to improve patients’ lifestyles (therefore their quality of life) can be evaluated, and, according to this, the frequency of visits can be scheduled. Moreover, information obtained with this way of data collection could be easily visualized, analyzed and interpreted by all the professionals involved in the patients’ care pathway.

The analysis conducted in this study also verified and measured the correlation between the professionals’ impressions (categorized in four dimensions) and patients’ data collected by the device. A positive correlation was recorded between the median sleep hours, the median number of steps taken by the patient, and the collaboration between healthcare professionals/patients. This information could be significant as greater involvement by the healthcare professional in the treatment relationship with the patient can influence the patient to develop proactive behavior. The aspect of collaboration between professionals and patients may lead to greater use of the device, thus increasing the monitoring time and greater data recording. The information collected by the device may help both the healthcare professional to carry out targeted and personalized interventions and users to monitor their lifestyle. This allows patients to manage their health by implementing interventions to maintain good health in aging. For diabetic subjects, it is crucial to limit the consequences of disease, and a fundamental aspect of pursuing this objective is to maintain a good level of physical activity that could be recordable and measurable through the wearable device.

A last interesting aspect emerges from the fact that all the GPs and two (out of three) nurses strongly agreed with the statement “I think that such monitoring is more useful if carried out in a context of integrated medicine rather than by the individual general practitioner”. From the latter, we can grasp the potential strength of integrated care groups, that is, by involving different healthcare professional profiles, to give chronic patients complete care to ensure a better quality of life to live longer in their familiar place.

### Strength and Limitations

To the best of our knowledge, this is the first study that assesses the acceptance of a non-medical device from the point of view of both patients and healthcare professionals. Furthermore, it emerges that integrated care groups could be the ideal setting to ensure patients’ complete monitoring of their lifestyle.

On the other hand, this study has several main limitations. First, the small sample size may undermine the reliability of observed results, even if it is a feasibility study. Second, the questionnaires used have not been validated yet. Therefore, it would be essential to implement structured and validated questionnaires to better assess the device’s acceptability for both patients and healthcare professionals. Third, an informative summary of the data collected had not been offered, but patients themselves (and the GPs) independently looked at the collected data. It is possible that offering such an informative summary of data may lead to a much higher level of acceptance. Finally, it is possible that having selected some of the patients who, by their nature, are more prone to use technology (if not already users of similar devices) led to a bias in the results observed. It would be helpful to propose a similar study in subjects who are not familiar with these novel technologies to verify if a good acceptance of the device can still be observed. Moreover, further studies are needed to assess devices’ acceptability in other clinical conditions and for longer periods of time.

## 5. Conclusions

Despite the limited number of subjects involved in this study, it was observed that there is a good acceptance level towards these non-medical devices, according to both patients’ and healthcare professionals’ impressions, even if these results should be interpreted carefully. The correlation between professionals’ impressions and patients’ willingness to improve their health is an important issue that should be more investigated, for example, by increasing the follow-up time and analyzing any changes in progress. Moreover, it should be underlined that such devices are non-medical ones, and they could be available for all subjects, potentially making patients’ monitoring possible without costs for the NHS, simply by checking the already captured data.

## Figures and Tables

**Table 1:** Socio-demographic and baseline characteristics of the whole study sample. Data are reported as median (I and III quartiles) for continuous variables and absolute number (percentage) for categorical variables.

Characteristic	*n* = 11
Age in years, median (IQR)	69 (67–72)
Sex, *n* (%)	
Male	10 (91%)
Female	1 (9%)
Marital status, *n* (%)	
Married	10 (91%)
Widowed	1 (9%)
Educational level	
Primary school	4 (36%)
Secondary school	7 (64%)
BMI, median (IQR)	28 (27.5–31.0)

Abbreviations: BMI, body mass index.

**Table 2:** Distribution of questionnaire scores according to patients’ educational level (**Panel A**) and the type of professional (**Panel B**). Data are reported as median (I and III quartiles).

Panel A				
	Patients’ Educational Level			
Dimensions (Scale Maximum Score)	Primary School (*n* = 4)	Secondary School (*n* = 7)	Combined (*n* = 11)	*p*
Perceived usefulness (25)	19.5 (18.0–20.3)	17.0(13.0–18.0)	18.0 (14.5–19.5)	0.165
Perceived ease-of-use (35)	26.5 (24.5–29.3)	25.0 (21.0–27.0)	25.0 (22.5–28.0)	0.370
Equipment characteristics (30)	28.6 (27.2–31.0)	26.0 (24.0–28.5)	27.2 (25.5–29.5)	0.279
Privacy concern (15)	11.00 (10.25–11.25)	11.0 (7.0–13.0)	11.0 (7.5–11.5)	0.719
Perceived risk (15)	4.5 (3.8–5.5)	8.0 (8.0–9.0)	8.0 (4.5–8.0)	0.033
Facilitating conditions (10)	4.0 (2.0–7.0)	7.0 (6.0–8.0)	6.0 (4.0–8.0)	0.466
Subjective norm (15)	10.0 (9.0–11.5)	9.0 (7.0–12.5)	9.0 (8.5–12.0)	0.594
**Panel B**				
	**Professional Type**			
	**Nurse** **(*n* = 3)**	**Physician** **(*n* = 3)**	**Combined** **(*n* = 6)**	***p***
Clinical usefulness (35)	25.0 (22.5–28.0)	27.0 (25.0–29.5)	26.0 (23.5–30.0)	0.573
Management usefulness (15)	10.0 (10.0–11.0)	11.0 (10.0–12.0)	10.5 (10.0–11.7)	0.852
Web interface ease-of-use (15)	11.0 (9.5–11.5)	10.0 (9.0–11.5)	10.5 (8.5–11.7)	1
Collaboration (10)	8.0 (7.5–9.0)	7.0 (7.0–7.5)	7.5 (7.0–8.0)	0.405

**Table 3:** Distribution of patients’ data collected by the device and their acceptance level (for patients that ended the study). Data are reported as median (I and III quartiles).

	Patients’ Educational Level			
Patients’ Variables	Acceptability: No (*n* = 3)	Acceptability: Yes (*n* = 7)	Combined (*n* = 10)	*p*
Median steps	3859 (1929–6170)	6987 (6075–8138)	6612 (4384–8302)	0.23
Median sleep hours	6.0 (6.0–6.0)	7.0 (6.5–7.0)	7.0 (6.0–7.0)	0.24
Median wearing time (hours)	17.0 (4.5–20.5)	24.0 (23.5–24.0)	24.0 (8.5–24.0)	0.33
Median heart rate	69 (67–71)	53 (51–55)	53 (52–66)	0.07

## Data Availability

The data presented in this study are available upon request from the corresponding author.

## Supplementary Materials

The following are available online at https://www.mdpi.com/article/10.3390/ijerph18189806/s1; Table S1. Description of dimensions related to technology acceptance (patients), Table S2. Description of dimensions related to technology acceptance (professionals), Table S3. Technology acceptance questionnaire, Table S4. Questionnaire for professionals.
