# Supplementary material for: Improving Healthy Aging by Monitoring Patients’ Lifestyle through a Wearable Device: Results of a Feasibility Study
Source: Int J Environ Res Public Health. 2021 Sep 17;18(18):9806. doi: 10.3390/ijerph18189806 (PMC8469467; doi:10.3390/ijerph18189806)
Supplement: Supplementary file 1 [file ijerph-18-09806-s001.zip › Table S1.pdf]

Table S1. Description of dimensions related to technology acceptance (patients) (from Puri et al [13]).

Dimensions	Description
**Perceived usefulness**	Perceived usefulness refers to improvements in one's job performance but in the context of this study; it was adapted to refer to the degree to which using a technology can help monitor older adults' health and support aging-in-place.
**Perceived ease of use**	Perceived ease of use has been established as a key indicator for user acceptance and is defined as "the degree to which a person believes that using a technology will be free from effort"
**Subjective norm**	Subjective norm is another dimension that is representative of user acceptance, and it is defined as the likelihood of recommending the use of the said technology to individuals who are influential in the lives of the technology user
**Facilitating conditions**	A review of literature revealed varying classifications of facilitating conditions. Facilitating factors are factors that can increase or decrease the effort required to use a technology such as availability, affordability, availability of training resources, and so on
**Privacy concerns**	Privacy concerns is a novel dimension in the framework and has been included because of the emergent tendency of smart device and technology manufacturers to use Internet communication protocols to store and analyse data in the cloud, rather than on the particular device.
**Perceived risks**	Perceived risks have been established to be influential to consumer behaviour and important when evaluating user acceptance of technology
**Equipment characteristics**	Finally, equipment characteristics that can influence the technology acceptance were deemed an important dimension and described as one of the major factors in another study
