# Supplementary material for: Improving Healthy Aging by Monitoring Patients’ Lifestyle through a Wearable Device: Results of a Feasibility Study
Source: Int J Environ Res Public Health. 2021 Sep 17;18(18):9806. doi: 10.3390/ijerph18189806 (PMC8469467; doi:10.3390/ijerph18189806)
Supplement: Supplementary file 1 [file ijerph-18-09806-s001.zip › Table S2.pdf]

Table S2. Description of dimensions related to technology acceptance (professionals).

| Dimensions | Description |
|---|---|
| **Clinical usefulness** | It investigates the perception of the usefulness of this type of monitoring in the IGM setting |
| **Management usefulness** | For management usefulness we intend to investigate the utility of use in practical management: time spent and resources. |
| **Web interface ease of use** | This dimension investigates the ease of viewing and collecting data recorded by the device |
| **Collaboration** | Collaboration means how much the healthcare professional feels involved in managing this type of monitoring and how much collaboration they perceive from the patient |
