# Supplementary material for: Improving Healthy Aging by Monitoring Patients’ Lifestyle through a Wearable Device: Results of a Feasibility Study
Source: Int J Environ Res Public Health. 2021 Sep 17;18(18):9806. doi: 10.3390/ijerph18189806 (PMC8469467; doi:10.3390/ijerph18189806)
Supplement: Supplementary file 1 [file ijerph-18-09806-s001.zip › Table S3.pdf]

Table S3. Technology Acceptance Questionnaire (from Puri et al [13]).

N° item	Corresponding dimension					Statement
						**1 = Strongly Disagree, 2 = Disagree, 3 = Neutral, 4 = Agree, 5 = Disagree**
1- PU	**1**	**2**	**3**	**4**	**5**	I think that monitoring my activity and health 24 hours a day, 7 days a week, can be a good thing
2 - PR	**1**	**2**	**3**	**4**	**5**	I was afraid that the device would discover a major health issue
3 - PC	**1**	**2**	**3**	**4**	**5**	I am comfortable with my health data being stored on the internet
4 - FC	**1**	**2**	**3**	**4**	**5**	I am able to get assistance from a friend of family member to use the device, if needed
5 -PEOU	**1**	**2**	**3**	**4**	**5**	I was able to wear the device easily without help from another person
6 -PEOU	**1**	**2**	**3**	**4**	**5**	I was able to remove the device easily without help from another person
7 -EC	**1**	**2**	**3**	**4**	**5**	I was able to perform my daily tasks as usual while wearing the device
8 -EC	**1**	**2**	**3**	**4**	**5**	The device was easily concealed underneath my clothing when worn
9 - EC	**1**	**2**	**3**	**4**	**5**	At times, I forgot I was wearing the device
10 - PR	**1**	**2**	**3**	**4**	**5**	I experienced skin irritations while wearing the device
11 -EC	**1**	**2**	**3**	**4**	**5**	The battery life of the device meets my expectations
12 -EC	**1**	**2**	**3**	**4**	**5**	The device’s smartphone application was easy to use
13 -PEOU	**1**	**2**	**3**	**4**	**5**	I find the device easy to use
14 -PEOU	**1**	**2**	**3**	**4**	**5**	I find the display of the device easy to read indoors

15PEOU	**1**	**2**	**3**	**4**	**5**	I find the display of the device easy to read outdoors
16 - EC	**1**	**2**	**3**	**4**	**5**	The device was pleasant to wear during the night
17 -EC	**1**	**2**	**3**	**4**	**5**	I was concerned that the device is not securely attached to me
18 - PEOU	**1**	**2**	**3**	**4**	**5**	I was able to put the device on in a reasonable amount of time
19 - PC	**1**	**2**	**3**	**4**	**5**	I had no concerns about my privacy while wearing the device
20 - PC	**1**	**2**	**3**	**4**	**5**	I am comfortable with my health data being shared with equipment manufacturers as long as it is shared anonymously
21 -FC	**1**	**2**	**3**	**4**	**5**	I have the knowledge necessary to use the device
22 - PU	**1**	**2**	**3**	**4**	**5**	I think using the device is a more efficient way to monitor my health than visiting my doctor to collect similar information
23 -PU	**1**	**2**	**3**	**4**	**5**	Wearing the device motivated me to be more active
24 - PU	**1**	**2**	**3**	**4**	**5**	I think using the device can help me improve my overall health
25 -PR	**1**	**2**	**3**	**4**	**5**	Wearing the device caused me to have joint pain
26 -PEOU	**1**	**2**	**3**	**4**	**5**	I was able to shower or bathe normally while wearing the device
27 -SN	**1**	**2**	**3**	**4**	**5**	I was embarrassed to wear the device in front of family membersEro imbarazzato dal fatto di indossare il device davanti ai miei familiari
28 -SN	**1**	**2**	**3**	**4**	**5**	My friends would encourage me to use this device
29 - SN	**1**	**2**	**3**	**4**	**5**	My family members would encourage me to use this device

30 -PU	**1**	**2**	**3**	**4**	**5**	I think using the device can let me live at home longer by monitoring my health around the clock
31 -EC	**1**	**2**	**3**	**4**	**5**	The ability to use the device in a variety of locations is important to me

Additional 6 multiple-choice questions

N° item	Corresponding dimension	Statement	Answers
32	NA	How useful did you find the information provided by the smart wearable device (such as step count, sleep data, heart rate) either on the wearable itself, or in the smartphone application?	a) very useful<br>b) somewhat useful<br>c) not very useful<br>d) not at all useful
33	NA	Would you use the device you used during the last 21 days to continue to monitor or track your physical activity or health?	a) yes<br>b) no
34	NA	Over the last 21 days, how often do you think you wore the smart wearable device?	a) never<br>b) at least 2 days per week<br>c) at least 4 days per week<br>d) at least 6 days per week<br>e) always
35	NA	How much would you be willing to pay for the device you wore during the last 21 days?	a) 0<br>b) 1-50<br>c) 51-100<br>d) 101-200<br>e) 201-300<br>f) 301-400
36	NA	Did you find yourself looking at your health data in the smartphone application more/less often after the first few days?	a) No, I looked at the health data consistently throughout the 21-day period

			b) Yes, I looked at the health data more often after the first few days of use c) Yes, I looked at the health data less often after the first few days of use<br><br>d) I did not look at my health or am not interested in monitoring it
37	NA	Do you consider yourself to be an active person?	a) yes<br><br>b) no

Legend of dimensions: PU, perceived usefulness; PEOU, perceived ease of use; EC, equipment characteristics; PC, privacy concern; PR, perceived risk; FC, facilitating conditions; SN, subjective norm; NA, not applicable.
