# Supplementary material for: Improving Healthy Aging by Monitoring Patients’ Lifestyle through a Wearable Device: Results of a Feasibility Study
Source: Int J Environ Res Public Health. 2021 Sep 17;18(18):9806. doi: 10.3390/ijerph18189806 (PMC8469467; doi:10.3390/ijerph18189806)
Supplement: Supplementary file 1 [file ijerph-18-09806-s001.zip › Table S4.pdf]

Table S4. Questionnaire for professionals.

Profession: **Age:**
Integrated Group Medicine:
Years of experience: **Years of experience in IGM:**

N° item						Statement
						**1 = Strongly Disagree, 2 = Disagree, 3 = Neutral, 4 = Agree, 5 = Disagree**
1-CU	**1**	**2**	**3**	**4**	**5**	I think that monitoring my patients' activity and health 24 hours a day, 7 days a week, can be a good thing in my clinical practice
2-CO	**1**	**2**	**3**	**4**	**5**	I feel active in monitoring my patient
3-CU	**1**	**2**	**3**	**4**	**5**	I think this kind of monitoring can be a support to the activities of MGI
4-WIEU	**1**	**2**	**3**	**4**	**5**	The website, where patients' data are stored, is easy to use
5-MU	**1**	**2**	**3**	**4**	**5**	I don't think this kind monitoring requires a lot of times to be done
6-CU	**1**	**2**	**3**	**4**	**5**	I think this kind of monitoring is more useful if it is done in a MDI setting rather than GP setting.
7-WIEU	**1**	**2**	**3**	**4**	**5**	I didn't have any difficulties in answering to patients' doubts and questions
8-CO	**1**	**2**	**3**	**4**	**5**	I felt that patients were available and collaborative in this type of monitoring
9-MU	**1**	**2**	**3**	**4**	**5**	In order to monitoring I think using a non-medical device is easier to accept for patients
10-CU	**1**	**2**	**3**	**4**	**5**	I think this kind of data are useful for the assistance provided to this type of patient
11-CU	**1**	**2**	**3**	**4**	**5**	I think the data collected from this type of monitoring is enough for the assistance provided to this type of patient
12-CU	**1**	**2**	**3**	**4**	**5**	I think the data collected from this type of monitoring is necessary for the assistance provided to this type of patient
13-CU	**1**	**2**	**3**	**4**	**5**	I think that monitoring my patients in this way can help me picking up early signs of any clinical problems / complications

<table>
<tr><td>14-MU</td><td>1</td><td>2</td><td>3</td><td>4</td><td>5</td><td>I think the data displayed may be a criterion for scheduling visits</td></tr>
<tr><td>15-WIEU</td><td>1</td><td>2</td><td>3</td><td>4</td><td>5</td><td>I find correspondence between the recorded data and the experiences reported by the patients</td></tr>
<tr><td>18</td><td colspan="5">What determined the frequency of the visualization of the collected data?</td><td>a) clinical picture of the patient
b) time available
c) a predetermined independent cadence of the patient</td></tr>
<tr><td>19</td><td colspan="5">How long would it take to visualize the data to be useful for clinical practice?</td><td></td></tr>
<tr><td>18</td><td colspan="5">What other types of patients could benefit from this type of monitoring?</td><td></td></tr>
</table>

Legend of dimensions: CU, clinical usefulness; MU, management usefulness; WIEU, web interface ease-of-use; COLL, collaboration.
